# Ultrasound-defined fatty liver and the risk of progression from preclinical to clinical obesity: a prospective cohort study

**DOI:** 10.3389/fnut.2026.1877518

**Published:** 2026-09-22

**Authors:** Haixiang Zheng, Peikai Huang, Guian Hong, Yuxian Wang, Zhenyu Huo, Yajing Liang, Hong Zheng, Yulong Lan, Shuohua Chen, Gianpaolo Vidili, Gavino Casu, Leonardo A. Sechi, Shouling Wu, Youren Chen

**Affiliations:** 1 Department of Cardiology, The Second Affiliated Hospital of Shantou University Medical College, Shantou, China; 2 Department of Biomedical Sciences, University of Sassari, Sassari, Italy; 3 School of Public Health, North China University of Science and Technology, Tangshan, China; 4 Department of Rheumatology and Immunology, Kailuan General Hospital, North China University of Science and Technology, Tangshan, China; 5 Department of Cardiology, Kailuan General Hospital, Tangshan, China; 6 Department of Medicine, Surgery, and Pharmacy, University of Sassari, Azienda Ospedaliero, Sassari, Italy; 7 Clinical and Interventional Cardiology, University Hospital, Azienda Ospedaliero, Sassari, Italy; 8 SC Microbiology and Virology, Azienda Ospedaliero University of Sassari, Sassari, Italy

**Keywords:** clinical obesity, Kailuan Study, organ dysfunction, preclinical obesity, prospective cohort study, ultrasound-defined fatty liver

## Abstract

**Background:**

According to the 2025 international consensus published in The Lancet Diabetes & Endocrinology, obesity can be conceptualized as a progressive disease with two stages: preclinical obesity, characterized by excess adiposity without established organ dysfunction, and clinical obesity, defined by the presence of obesity-related organ damage. However, determinants of the transition from preclinical to clinical obesity remain poorly understood. Fatty liver, a common manifestation of metabolic dysfunction, may represent an early indicator of this progression.

**Methods:**

We analyzed 18,955 adults with preclinical obesity but without clinical obesity at baseline from the Kailuan Study. Fatty liver was diagnosed by abdominal ultrasound and categorized as mild or moderate-to-severe. Clinical obesity was defined as the development of at least one obesity-related organ dysfunction during follow-up. Weighted Cox regression was used to estimate the average association between baseline fatty liver and incident clinical obesity after multivariable adjustment, while extend Cox models with time-varying coefficients were used to evaluate changes in the association over follow-up.

**Results:**

During a median follow-up of 7.8 years, 11,463 participants developed clinical obesity. Ultrasound-defined fatty liver was independently associated with an increased risk of progression to clinical obesity (fully adjusted HR: 1.59, 95% CI: 1.50–1.68), with a severity-dependent gradient (mild: HR 1.55, 95% CI: 1.45–1.65; moderate-to-severe: HR 1.70, 95% CI: 1.59–1.82). Women with moderate-to-severe fatty liver had the highest risk (HR: 1.77, 95% CI: 1.56–2.01). Results were consistent in sensitivity analyses. Hypertension, obstructive sleep apnea syndrome, and hyperglycemia-dyslipidemia syndrome were the most common organ dysfunctions contributing to clinical obesity.

**Conclusions:**

Ultrasound-defined fatty liver is associated with a significantly increased risk of progressing from preclinical to clinical obesity. These findings suggest that ultrasound-defined fatty liver may help identify individuals with preclinical obesity who are at increased risk of progressing to clinical obesity and may support targeted metabolic surveillance.

## Introduction

According to epidemiological surveillance data from the World Health Organization, the global prevalence of obesity in adults has more than doubled since 1990 and will reach 16% in 2022 (1). China is facing a similarly critical challenge. The Report on Nutrition and Chronic Disease Status of Chinese Residents (2020) shows that the prevalence of overweight and obesity among Chinese adults in 2018 was 34.3% and 16.4%, respectively (2). If current trends continue without effective interventions, the combined prevalence of overweight and obesity in China is expected to rise to 70.5% by 2030 (3), with direct medical costs associated with obesity estimated at over USD 61 billion (4). Epidemiologic studies have consistently shown a significant positive association between obesity and the incidence of type 2 diabetes, hypertension, myocardial infarction (MI), cerebrovascular events, and various malignancies (5–9). Overall, these obesity-related diseases contribute significantly to premature mortality (10). Despite its widespread clinical use, body mass index (BMI) has notable limitations as a primary diagnostic criterion for obesity. BMI does not distinguish between fat mass and lean mass (e.g., muscle and bone), overlooks the heterogeneity of fat distribution and does not adequately reflect metabolic health status (11). These limitations may lead to misclassification of the health risks associated with obesity, particularly in individuals with metabolically healthy obesity (MHO) or normal weight obesity with metabolic dysfunction (12).

Recently, the Lancet Commission on Diabetes and Endocrinology redefined the clinical classification of obesity and introduced the concepts of “preclinical obesity” and “clinical obesity” (13). According to this new framework, clinical obesity is characterized not only by anthropometric criteria such as BMI, waist circumference (WC), waist-to-hip ratio (WHR) and waist-to-height ratio (WHtR), but also by the presence of at least one obesity-related chronic non-communicable disease associated with organ dysfunction, such as liver fibrosis. In contrast, preclinical obesity refers to individuals who meet the anthropometric thresholds for obesity but do not have clinical indicators of end-organ damage. Nevertheless, a subset of this population may already have hepatic steatosis even if there are no obvious clinical complications.

Metabolic dysfunction–associated steatotic liver disease (MASLD), defined as hepatic steatosis—most commonly diagnosed by ultrasonography—in the presence of metabolic dysfunction, affects nearly 30% of adults worldwide and is now a leading cause of chronic liver disease (14). There is evidence that MASLD not only impairs liver function but is also an independent risk factor for extrahepatic complications. A meta-analysis that included 20 cohort studies and more than 78,000 patients with non-alcohol fatty liver disease (NAFLD) diagnosed by ultrasound showed a 37% increased risk of cardiovascular disease (CVD) compared to individuals without NAFLD (15). Similarly, in a large-scale Korean cohort study of 111,492 adults with no previous CVD, those with ultrasound-diagnosed NAFLD had a 54% higher risk of MI (16). These results show that even simple steatosis detected by ultrasound is not a benign condition, but rather a predictor of significantly increased cardiovascular morbidity.

MHO refers to obesity without conventional metabolic abnormalities. Although related, MHO and preclinical obesity are not interchangeable: MHO is defined mainly by metabolic status, whereas preclinical obesity requires excess adiposity without established obesity-related organ dysfunction or substantial limitations in daily activities. Despite this apparently favorable profile, individuals with MHO remain at increased cardiovascular risk and may progress to metabolic syndrome (MetS), a cluster of abdominal obesity, elevated blood pressure, dysglycemia, and dyslipidemia (17–19). These findings suggest that markers capable of identifying higher-risk individuals before overt metabolic deterioration are needed.

Fatty liver was selected as the exposure of interest because it reflects ectopic hepatic fat accumulation and may capture metabolic risk beyond BMI and conventional cardiometabolic markers. In previous studies using the NAFLD terminology, ultrasound-defined NAFLD was reported in 7.14% of metabolically healthy normal-weight individuals and 34.8% of those with MHO in a Chinese population (20). In the ATTICA cohort, NAFLD was present in approximately 29%−39% of participants with MHO and was associated with subsequent transition to metabolically unhealthy obesity (21). Although the criteria used to define MHO in these studies differed from those used to define preclinical obesity, these findings indicate that hepatic steatosis is common among apparently metabolically healthy individuals and may help identify those at greater risk of metabolic deterioration. Given the noninvasive and widely available nature of abdominal ultrasonography, ultrasound-defined fatty liver may provide a practical imaging phenotype for early risk stratification. Therefore, the present study investigated whether the impact of fatty liver was associated with progression from preclinical to clinical obesity.

## Methods

### Study design and participants

The Kailuan Study is a prospective cohort study conducted in the Kailuan community in Tangshan City, Hebei Province, China. The detailed study design and methodology have already been described (22, 23). Between 2006 and 2007, Kailuan General Hospital and its 10 affiliated hospitals conducted health surveys among current and retired employees of the Kailuan Group. A total of 101,510 participants aged 18–98 years, including 81,110 men, underwent standardized questionnaires, clinically examinations, and laboratory testing, with follow-up examinations conducted every 2 years. We first excluded participants with missing data for at least two of the four anthropometric measurements (BMI, WC, WHR, or WHtR; *n* = 2,140) and those with secondary hypertension (*n* = 139), leaving 99,231 participants. We then excluded participants who did not meet the anthropometric criteria for obesity (*n* = 45,297) and those with clinical obesity at baseline (*n* = 34,458), yielding 19,476 participants with preclinical obesity. Finally, we excluded participants with missing ultrasound data on hepatic steatosis (*n* = 111), a fibrosis 4 index (FIB-4) score >3.25 (*n* = 62), or missing data on fasting blood glucose, triglycerides, high-density lipoprotein cholesterol (HDL-c), creatinine, systolic blood pressure, diastolic blood pressure, or alanine aminotransferase (ALT; *n* = 348). Consequently, 18,955 participants with preclinical obesity were included in the final analysis and followed longitudinally (Figure 1).

**Figure 1:**
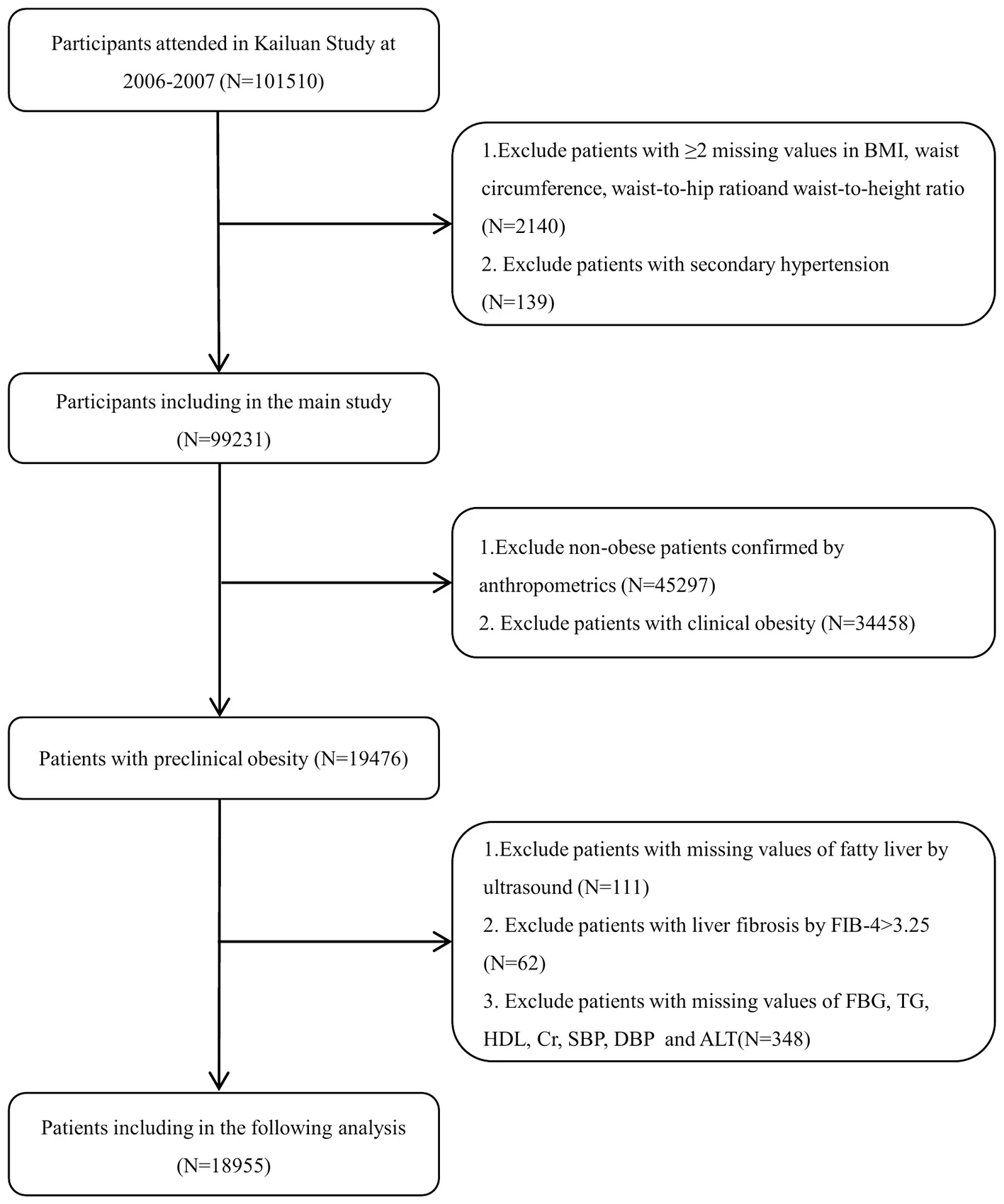
Flow chart of participant selection.

### Data collection and assessment of covariates

Trained staff administered structured questionnaires to collect information on demographic characteristics and lifestyle factors, including age, sex, marital status, education level, physical activity, current smoking, current alcohol consumption. Data on medical history (diabetes, MI, cancer) and medication use (lipid-lowering and hypoglycemic agents) were also collected. The definitions of these variables were consistent with those of previously published studies (24). BMI was calculated as weight (kg) divided by height squared (m^2^). WHtR was calculated as WC (cm) divided by height (cm) and WHR was calculated as WC (cm) divided by hip circumference (cm). Centralized biochemical profiling utilized a Hitachi 747 automated analyzer (Hitachi Ltd., Tokyo, Japan) to measure triglycerides, total cholesterol (TC), HDL-c, low-density lipoprotein cholesterol (LDL-c, calculated via the Friedewald Formula), uric acid, high-sensitivity C-reactive protein (hs-CRP), serum creatinine, fasting blood glucose, and ALT.

### Assessment of fatty liver

To assess hepatic steatosis, abdominal ultrasonography was performed using a PHILIPS HD15 color Doppler ultrasound system equipped with a convex transducer (1–5 MHz) and operated by sonographers with more than 5 years of clinical experience who had received standardized training. The diagnosis required meeting at least two of the following criteria: (a) diffuse enhancement of liver echogenicity compared to renal parenchyma; (b) significant attenuation of ultrasound beams; (c) suboptimal visualization of intrahepatic vascular structures. The severity of hepatic steatosis was categorized into three grades: mild (diffuse fine echogenicity in hepatic parenchyma), moderate (diffuse fine echogenicity with partially obscured but distinguishable intrahepatic vascular boundaries and diaphragmatic visualization), and severe (diffuse fine echogenicity with indistinct intrahepatic vascular boundaries and diaphragmatic visualization that could not be differentiated) (25). Liver fibrosis was assessed using the FIB-4, which is calculated as follows: FIB-4 index = [age × aspartate aminotransferase (AST)]/(platelet count × √ALT). To ensure diagnostic consistency, all ultrasound images were reviewed and confirmed jointly by two experienced ultrasonographers. In addition, a random subset of images was reassessed by independent ultrasound experts to validate the findings. The sonographers were blinded to the participants' clinical and laboratory data. Due to the limited number of severe fatty liver cases, moderate and severe steatosis were combined into a single category (“moderate-to-severe steatosis”) to increase the statistical power of the analysis.

### Definition of preclinical and clinical obesity

Preclinical obesity was defined as the absence of limitation of daily activity or organ dysfunction together with at least one of the following anthropometric criteria: (1) BMI > 40 kg/m^2^; (2) BMI ≥ 28 kg/m^2^ in conjunction with one of the following elevated parameters —WC, WHR or WHtR; (3) two of WC, WHR or WHtR simultaneously elevated (Supplementary Table S1). Clinical obesity was defined as the presence of preclinical obesity associated with at least one organ dysfunction according to the criteria proposed by the Commission on Diabetes and Endocrinology and published in The Lancet (13) (Supplementary Table S2).

### Long-term follow-up and outcome assessment

The baseline status of participants in this cohort study was determined by their initial participation in the Kailuan Health Survey in 2006, and individuals who met the criteria for preclinical obesity were purposively selected. The primary outcome was defined as the occurrence of new clinical obesity. Follow-up time was calculated from baseline to the first diagnosis of clinical obesity as the endpoint and ended on December 31, 2021.

### Statistical analysis

All statistical analyses were performed with SAS version 9.4 (SAS Institute Inc., Cary, NC, USA) and R Studio version 4.3.3 (R Foundation for Statistical Computing, Vienna, Austria). Continuous variables with skewed distributions were summarized as medians with interquartile ranges (IQR), while normally distributed variables were expressed as means ± standard deviation (SD). Categorical variables were presented as frequencies and percentages. Comparisons of continuous variables were performed using the Kruskal-Wallis test or one-way analysis of variance (ANOVA), and categorical variables were compared using the chi-square test. The cumulative incidence of clinical obesity in participants with and without fatty liver was estimated using the Kaplan–Meier method, and differences between groups were assessed using the log-rank test. Since the proportional hazards assumption was violated (*P* < 0.05), weighted Cox regression based on the method of Schemper et al. (26) was used to estimate hazard ratios (HRs) representing the average association over the follow-up period. Model 1 was adjusted for age and sex. Model 2 further adjusted for marital status, education level, physical activity, current smoking, current alcohol consumption, heart rate, hs-CRP, and TC. Model 3 additionally adjusted for use of hypoglycemic and lipid-lowering medications, as well as history of MI and cancer. Covariate were selected a priori based on previous literature, clinical knowledge, and their potential associations with both ultrasound-defined fatty liver and incident clinical obesity. No automated or stepwise variable-selection procedure was used. Multicollinearity among covariates in the fully adjusted model was assessed using variance inflation factor (VIF), with a VIF < 5 indicating no substantial multicollinearity (Supplementary Table S3). We also stratified the fatty liver group into mild and moderate-to-severe subgroups and conducted multivariable weighted Cox regression with the same covariate adjustments. To further characterize temporal variation in the associations, extended Cox models were fitted with time-varying coefficients for baseline fatty liver status and fatty liver severity, allowing the corresponding HRs to vary over follow-up time (27). The resulting time-varying HRs were plotted against follow-up time. Subgroup analyses were performed by age group (>65 vs. ≤ 65 years), sex, physical activity, current alcohol consumption, and heart rate (>60 vs. ≤ 60 bpm). Subgroup and interaction analyses were exploratory, with nominal *P* values reported without adjustment for multiple comparisions. Several sensitivity analyses were performed to assess the robustness of the results: (1) exclusion of participants who developed clinical obesity within the first year of follow-up to minimize reverse causality; (2) exclusion of participants taking blood glucose-lowering or lipid-lowering agents; (3) exclusion of participants with preclinical obesity with a BMI < 24.0 kg/m^2^ to avoid potential misclassification; and (4) re-inclusion of the 62 participants excluded solely because of a FIB-4 score >3.25. Finally, we analyzed the distribution and types of organ dysfunction associated with clinical obesity and compared these between the groups with and without fatty liver. The results were visualized using bar charts. A two-sided *P*-value < 0.05 was considered statistically significant.

## Results

### Baseline characteristics

As shown in Table 1, a total of 18,955 participants with preclinical obesity at baseline were included in the analysis. The mean age of the study population was 50.60 ± 12.06 years, and 75.0% were male (*n* = 14,218). Of the included participants, 12,460 (65.7%) had no ultrasound-defined fatty liver and served as the reference group, whereas 6,495 (34.3%) had fatty liver, including 4,597 (24.3%) with mild fatty liver and 1,898 (10.0%) with moderate-to-severe fatty liver. Compared with participants without fatty liver, those with fatty liver were younger, had a higher proportion of men and current smokers, and had significantly higher levels of systolic blood pressure, diastolic blood pressure, TC, triglyceride, LDL-c, fasting glucose, uric acid, creatinine, ALT and hs-CRP (all *P* < 0.01). Further comparisons revealed that individuals with moderate-to-severe fatty liver had more pronounced metabolic and inflammatory abnormalities than those with mild fatty liver, including significantly elevated fasting glucose, triglyceride, uric acid, hs-CRP, ALT levels and a higher rate of lipid-lowering medications, as well as significantly lower HDL-c levels (all *P* < 0.01).

**Table 1:** Baseline characteristics of participants with preclinical obesity according to ultrasound-defined fatty liver status.

Indicator type	Variables	Total (*n* = 18,955)	Without fatty liver (*n* = 12,460)	Fatty liver		*P*-value
				Mild (*n* = 4,597)	Moderate-severe (*n* = 1,898)	
Sociodemographic	Ages, years	50.60 ± 12.06	51.09 ± 12.20	49.91 ± 11.60	49.05 ± 12.01	< 0.01
	Male, *n* (%)	14,218 (75.0)	9,102 (73.0)	3,617 (78.7)	1,499 (79.0)	< 0.01
	Have spouse, *n* (%)	18,281 (96.4)	12,057 (96.8)	4,417 (96.1)	1,807 (95.2)	< 0.01
	High school diploma or above, *n* (%)	3,365 (17.8)	1,872 (15.0)	1,063 (23.1)	430 (22.7)	< 0.01
Anthropometric	BMI, kg/m^2^	25.88 ± 3.24	25.00 ± 3.04	27.20 ± 2.77	28.45 ± 3.10	< 0.01
	WC, cm	92.59 ± 7.63	91.79 ± 7.76	93.55 ± 6.80	95.59 ± 7.69	< 0.01
	WHR	0.93 ± 0.07	0.94 ± 0.07	0.92 ± 0.06	0.93 ± 0.06	< 0.01
	WHtR	0.55 ± 0.04	0.55 ± 0.04	0.56 ± 0.04	0.57 ± 0.05	< 0.01
Vital signs	Heart rate, beats/min	72.65 ± 9.41	72.59 ± 9.44	72.69 ± 9.31	72.98 ± 9.45	0.23
	SBP, mmHg	119.04 ± 10.49	118.42 ± 10.70	119.86 ± 10.10	121.10 ± 9.53	< 0.01
	DBP, mmHg	77.21 ± 6.49	76.71 ± 6.73	78.02 ± 6.03	78.73 ± 5.53	< 0.01
Lifestyle	Physical activity, *n* (%)	2,124 (11.2)	1,370 (11.0)	523 (11.4)	231 (12.2)	0.29
	Smoke, *n* (%)	4,975 (26.2)	3,004 (24.1)	1,420 (30.9)	551 (29.0)	< 0.01
	Drink, *n* (%)	2,712 (14.3)	1,651 (13.3)	740 (16.1)	321 (16.9)	< 0.01
Blood biomarkers	TC, mmol/L	4.91 ± 1.06	4.86 ± 1.03	5.03 ± 1.05	4.94 ± 1.24	< 0.01
	TG, mmol/L	1.28 (0.92–1.86)	1.16 (0.84–1.62)	1.51 (1.09–2.24)	1.71 (1.23–2.63)	< 0.01
	LDL-C, mmol/L	2.21 (1.68–2.73)	2.17 (1.62–2.70)	2.29 (1.81–2.76)	2.30 (1.85–2.74)	< 0.01
	HDL-C, mmol/L	1.52 ± 0.39	1.53 ± 0.40	1.50 ± 0.38	1.47 ± 0.39	< 0.01
	FBG, mmol/L	5.20 ± 1.26	5.14 ± 1.24	5.32 ± 1.25	5.33 ± 1.35	< 0.01
	Uric acid, μmol/L	288.98 ± 82.72	275.87 ± 77.90	309.40 ± 83.52	325.60 ± 90.16	< 0.01
	Creatinine, μmol/L	88.08 ± 26.34	87.39 ± 27.41	88.06 ± 21.44	92.53 ± 29.32	< 0.01
	hs-CRP, mg/L	0.87 (0.30–2.48)	0.70 (0.26–2.12)	1.10 (0.46–3.00)	1.31 (0.58–3.21)	< 0.01
	ALT, U/L	18.0 (13.0–26.0)	16.6 (12.0–22.7)	21.0 (16.0–29.2)	26.1 (19.0–40.0)	< 0.01
History of disease	Diabetes Mellitus, *n* (%)	838 (4.4)	481 (3.9)	245 (5.3)	112 (5.9)	< 0.01
	MI, *n* (%)	150 (0.8)	90 (0.7)	48 (1.0)	12 (0.6)	0.08
	Cancers, *n* (%)	60 (0.3)	31 (0.3)	20 (0.4)	9 (0.5)	0.07
History of drugs	Hypoglycemic drugs, *n* (%)	209 (1.1)	122 (1.0)	62 (1.4)	25 (1.3)	0.08
	Lipid-lowering drugs, *n* (%)	6,275 (33.1)	3,451 (27.7)	1,885 (41.0)	939 (49.5)	< 0.01

Values are presented as mean ± SD or median (IQR) for continuous variables and number (percentage) for categorical variables. Analyses were based on available observations. BMI, WC, WHR, and WHtR were available for 18,944, 18,955, 18,643, and 18,953 participants, respectively. No missing values were imputed.

ALT, alanine aminotransferase; DBP, diastolic blood pressure; FBG, fasting blood glucose; hs-CRP, high-sensitivity C-reactive protein; HDL-c, high-density lipoprotein cholesterol; IQR, interquartile range; LDL-c, low-density lipoprotein cholesterol; MI, myocardial infarction; SBP, systolic blood pressure; SD, standard deviation; TC, total cholesterol; TG, triglyceride.

### Association between fatty liver and incident clinical obesity

Over a median follow-up period of 7.8 years (range: 3.6–13.8 years), a total of 11,463 participants with preclinical obesity developed clinical obesity. The incidence rate (IR) of clinical obesity was higher in participants with fatty liver than in participants without fatty liver. Kaplan–Meier curves showed that individuals with fatty liver had a significantly higher cumulative incidence of clinical obesity than those without fatty liver (log-rank *P* < 0.0001; Figure 2a). After adjustment for age and sex, individuals with preclinical obesity with fatty liver had a significantly increased risk of developing clinical obesity (HR = 1.66; 95% CI: 1.58–1.75; *P* < 0.001). In the fully adjusted multivariable model, the HR remained significant (HR = 1.59; 95% CI: 1.50–1.68; *P* < 0.001; Table 2). As fatty liver status may vary over time and influence the outcome, we performed a time-dependent Cox regression analysis for validation (Supplementary Table S4). In the multivariable-adjusted extended Cox model with a time-varying coefficient for baseline fatty liver status, the HR for incident clinical obesity was HR = 1.74 (95% CI: 1.62–1.86; *P* < 0.001) at the end of follow-up. Although the estimated HR decreased by approximately 1.9% per year, it remained above 1 throughout follow-up (Figure 3a).

**Figure 2:**
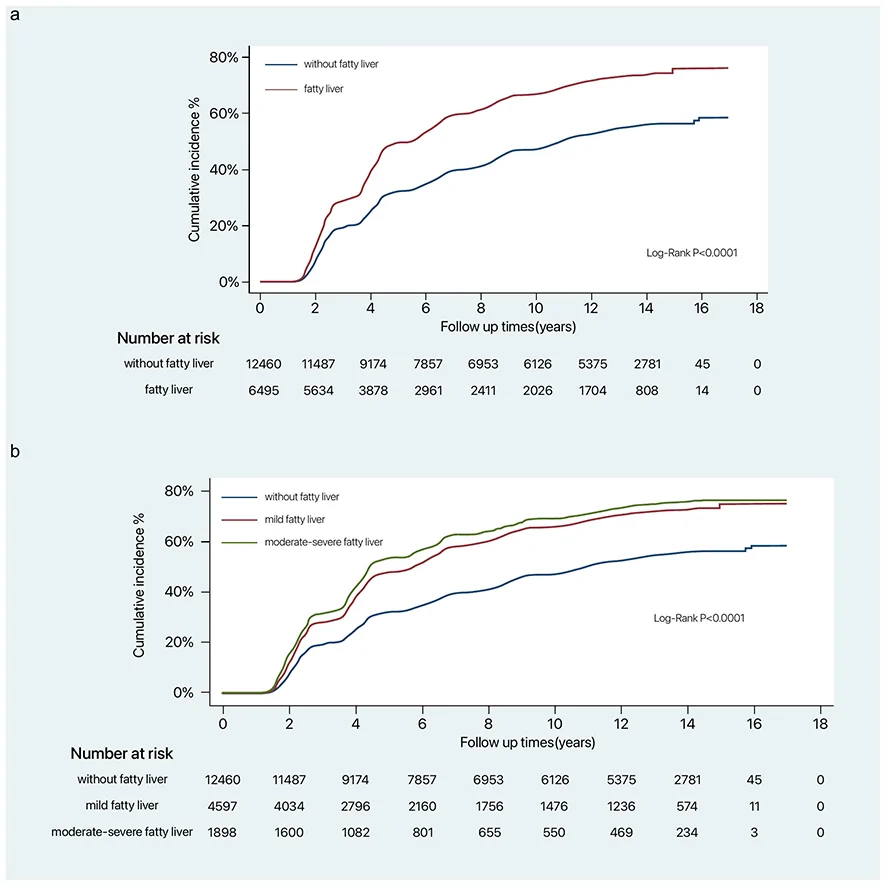
Cumulative incidence of incident clinical obesity according to baseline fatty liver status and severity. **(a)** Kaplan–Meier curves showing the cumulative incidence of new-onset clinical obesity in individuals with fatty liver (red line) compared with those without fatty liver (blue line) during up to 16 years of follow-up. **(b)** Kaplan–Meier curves showing the cumulative incidence of new-onset clinical obesity stratified by fatty liver severity: no fatty liver (blue), mild fatty liver (red), and moderate-to-severe fatty liver (green) during up to 16 years of follow-up. For both panels, the number at risk in each group is displayed below the x-axis. Participants with fatty liver (overall and by severity) exhibited a significantly higher cumulative incidence than those without fatty liver (Log-Rank *P* < 0.0001).

**Table 2:** Hazard ratios of incident clinical obesity using weighted Cox regression.

Measure	Without fatty liver	Fatty liver
Case/total	6,769/12,460	4,694/6,495
IR	61.12	104.00
Model 1	1 (reference)	1.66 (1.58–1.75)
Model 2	1 (reference)	1.61 (1.53–1.70)
Model 3	1 (reference)	1.59 (1.50–1.68)

Values are presented as cases/total, incidence rates (IRs; per 1,000 person-years), and hazard ratios (HRs) with 95% confidence intervals (CIs), with participants without fatty liver serving as the reference group. HRs were estimated using weighted Cox regression and represent average association over the follow-up period in the presence of non-proportional hazards. Model 1: adjusted for age sex; Model 2: adjust for education level, physical activity, current smoke, current drink, marital status, heart rate, hs-CRP, TC on the basis of model 1; Model 3: included variables in model 2 and further adjusted for history of myocardial infarction and cancers, hypoglycemic drugs and lipid-lowering drugs. All exposure and covariate values were obtained at baseline. CI, confidence interval; HR, hazard ratio; IR, incidence rate; hs-CRP, high-sensitivity C-reactive protein; TC, total cholesterol.

**Figure 3:**
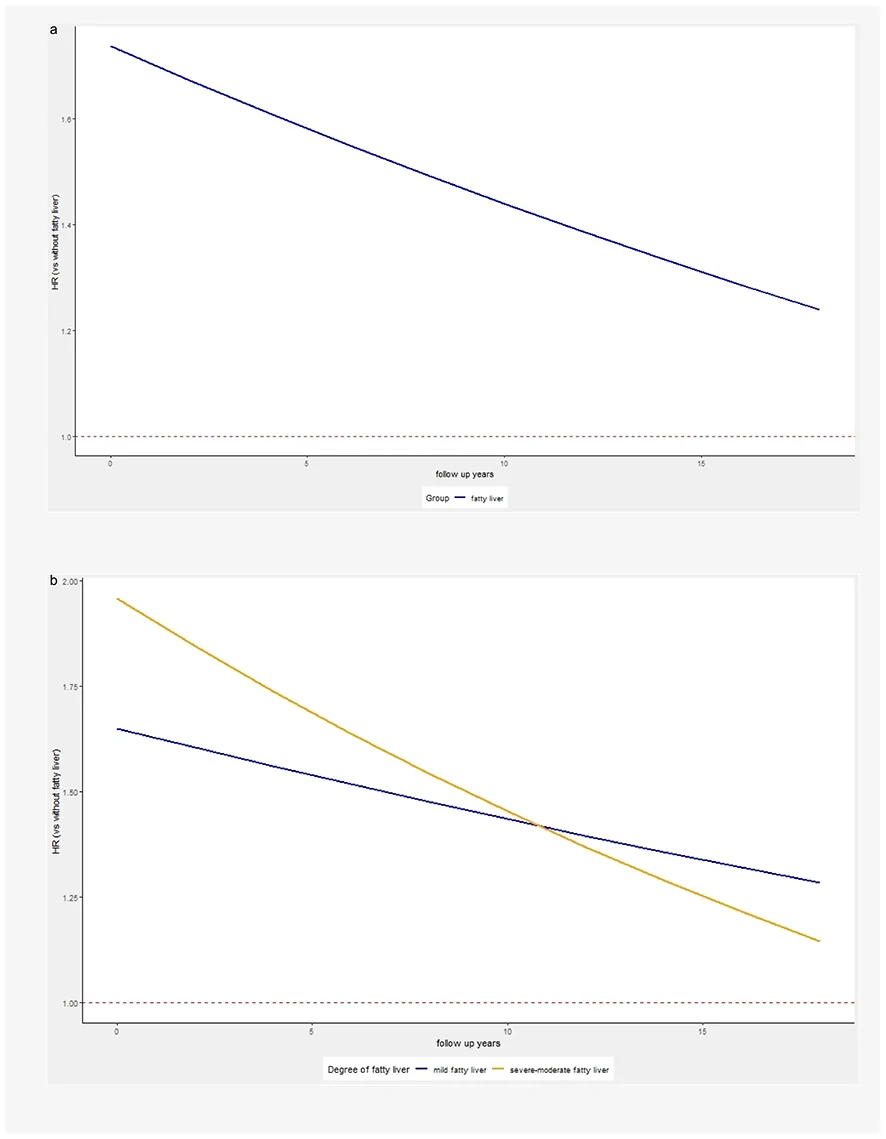
Time-varying hazard ratios for incident clinical obesity according to baseline fatty liver status and severity. **(a)** Time-varying hazard ratio (HR) for incident clinical obesity associated with baseline fatty liver, using participants without fatty liver at baseline as the reference group. The blue line represents the estimated HR over follow-up. **(b)** Time-dependent HRs for incident clinical obesity according to baseline fatty liver severity. The blue line represents mild fatty liver, and the yellow line represents moderate-to-severe fatty liver, with participants without fatty liver at baseline serving as the reference group. In both panels, the dashed horizontal line indicates the null value (HR = 1.0). The curves were derived from extended Cox models allowing the coefficients for baseline fatty liver status and severity to vary over follow-up time. HR, hazard ratio.

### Association between the severity of fatty liver and incident clinical obesity

To further explore the association between the severity of fatty liver and the incidence of clinical obesity, participants with fatty liver were categorized into mild and moderate-to-severe groups based on ultrasound diagnosis (severe and moderate steatosis were combined due to the limited number of severe cases to avoid bias). The results showed a dose-response relationship between the severity of fatty liver and the risk of clinical obesity incidence. Compared to participants without fatty liver, participants with mild and moderate-to-severe fatty liver had a significantly higher risk of clinical obesity, with HRs of 1.60 (95% CI: 1.52–1.71) and 1.79 (95% CI: 1.67–1.91), respectively, after adjustment for age and sex (*P* < 0.001). This trend also persisted in the fully adjusted multivariable model (Table 3). Kaplan–Meier curves showed that the cumulative incidence of clinical obesity was highest in participants with moderate-to-severe fatty liver, followed by participants with mild fatty liver and participants without fatty liver (log-rank *P* < 0.0001; Figure 2b). The time-dependent Cox regression analysis confirmed these results (Supplementary Table S5). In the corresponding extended Cox model with time-varying coefficients for baseline fatty liver severity, the HRs for incident clinical obesity at the end of follow-up were 1.65 (95% CI: 1.53–1.78) for mild fatty liver and 1.96 (95% CI: 1.76–2.18) for moderate-to-severe fatty liver (both *P* < 0.001). Although the estimated HRs decreased annually by 1.4% and 3.0%, respectively, both remained above 1 throughout follow-up (Figure 3b).

**Table 3:** Incidence rates and hazard ratios for incident clinical obesity according to baseline fatty liver status and severity.

Measure	Without fatty liver	Fatty liver	
		Mild	Moderate-to-severe
Case/total	6,769/12,460	3,275/4,597	1,419/1,898
IR	61.12	100.76	112.34
Model 1	1 (reference)	1.60 (1.52–1.71)	1.79 (1.67–1.91)
Model 2	1 (reference)	1.57 (1.47–1.66)	1.74 (1.63–1.86)
Model 3	1 (reference)	1.55 (1.45–1.65)	1.70 (1.59–1.82)

Values are shown as cases/total, incidence rates (IRs; per 1,000 person-years), and hazard ratios (HRs) with 95% confidence intervals (CIs), using participants without fatty liver at baseline serving as the reference group. HRs were estimated using weighted Cox regression and represent the average associations over the follow-up period in the presence of non-proportional hazards All exposure and covariate values were obtained at baseline. Model 1: adjusted for age sex; Model 2: adjust for education level, physical activity, current smoke, current drink, marital status, heart rate, high-sensitivity C-reactive protein (hs-CRP), TC (total cholesterol) on the basis of model 1; Model 3: included variables in model 2 and further adjusted for history of myocardial infarction and cancers, hypoglycemic drugs and lipid-lowering drugs. CI, confidence interval; HR, hazard ratio; hs-CRP, high-sensitivity C-reactive protein; IR, incidence rate; TC, total cholesterol.

### Subgroup and sensitivity analyses

The results of the subgroup analysis are shown in Figures 4, 5. Exploratory subgroup analyses suggested nominal evidence of interaction by sex for the association between fatty liver and incident clinical obesity (*P* for interaction = 0.04), with a stronger association observed in women (HR = 1.59; 95% CI: 1.47–1.73; *P* < 0.001). For moderate-to-severe fatty liver, nominal evidence of interaction by sex was also found (*P* for interaction = 0.02), with a stronger association in women (HR = 1.77; 95% CI: 1.56–2.01; *P* < 0.001).A potential interaction by heart rate was observed for overall fatty liver status (*P* for interaction = 0.04), whereas no corresponding interaction was observed in the analyses stratified by fatty liver severity. Several sensitivity analyses confirmed the robustness of the main results, including: (1) exclusion of participants who developed clinical obesity within the first year of follow-up; (2) exclusion of users of blood glucose-lowering or lipid-lowering medications; (3) exclusion of participants with a BMI < 24.0 kg/m^2^ at baseline; and (4) re-inclusion of the 62 participants originally excluded because of a FIB-4 score > 3.25. All sensitivity results were consistent with the primary analysis (Supplementary Tables S6–S9). Reliable criterion-specific HRs could not be estimated because of the very small BMI >40 kg/m^2^ subgroup and incomplete component-level anthropometric data.

**Figure 4:**
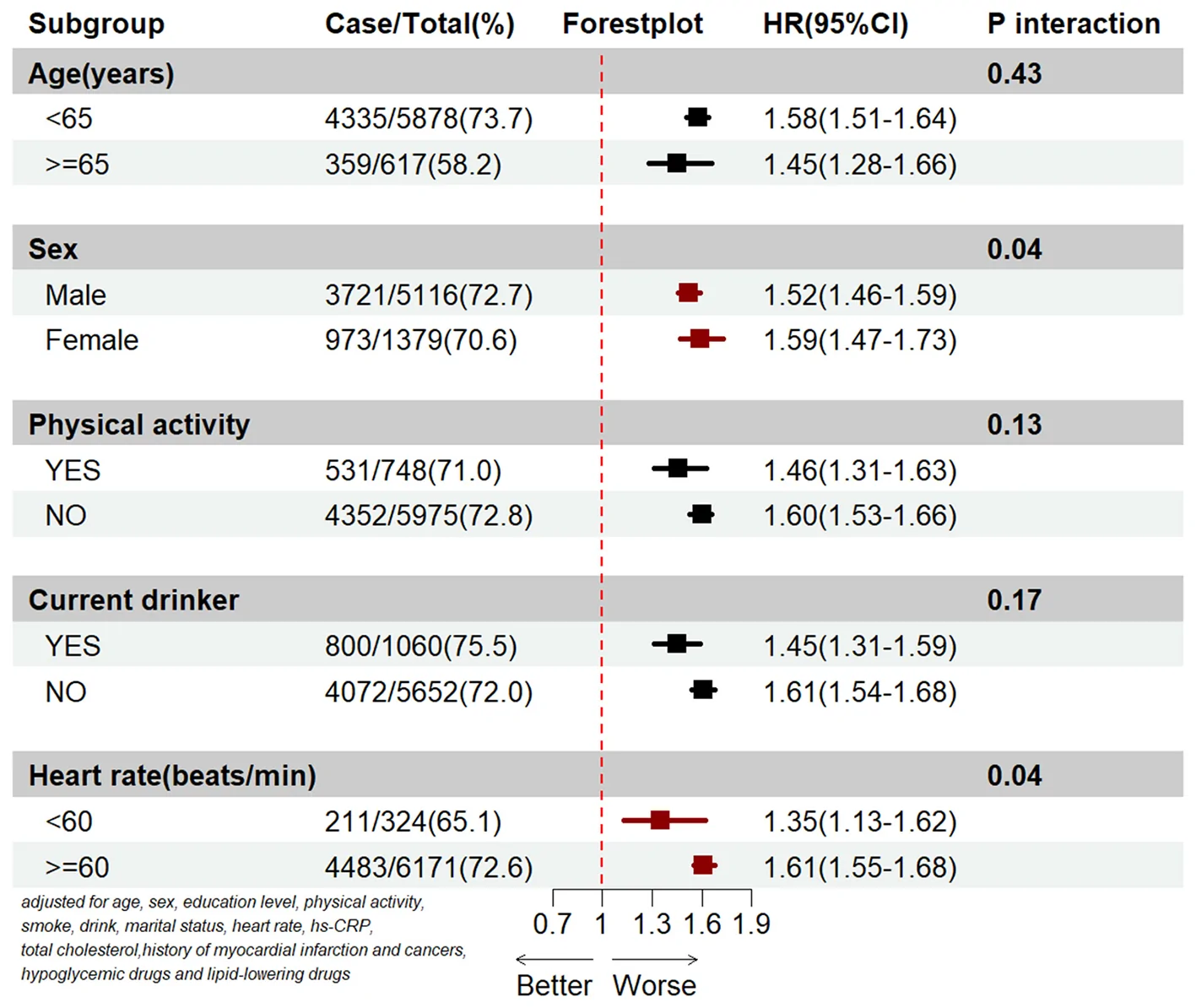
Subgroup analyses for the association between baseline ultrasound-defined fatty liver and incident clinical obesity. Forest plot shows hazard ratios (HRs) and 95% confidence intervals (CIs) comparing participants with fatty liver to those without fatty liver across prespecified subgroups (age, sex, physical activity, current drinking, and resting heart rate). Squares indicate point estimates and horizontal bars the 95% CIs; the vertical dashed line marks HR = 1.0. Numbers at left are cases/total (%). *P* for interaction was obtained from multiplicative interaction terms in Cox models. CI, confidence interval; HR, hazard ratio; hs-CRP, high-sensitivity C-reactive protein.

**Figure 5:**
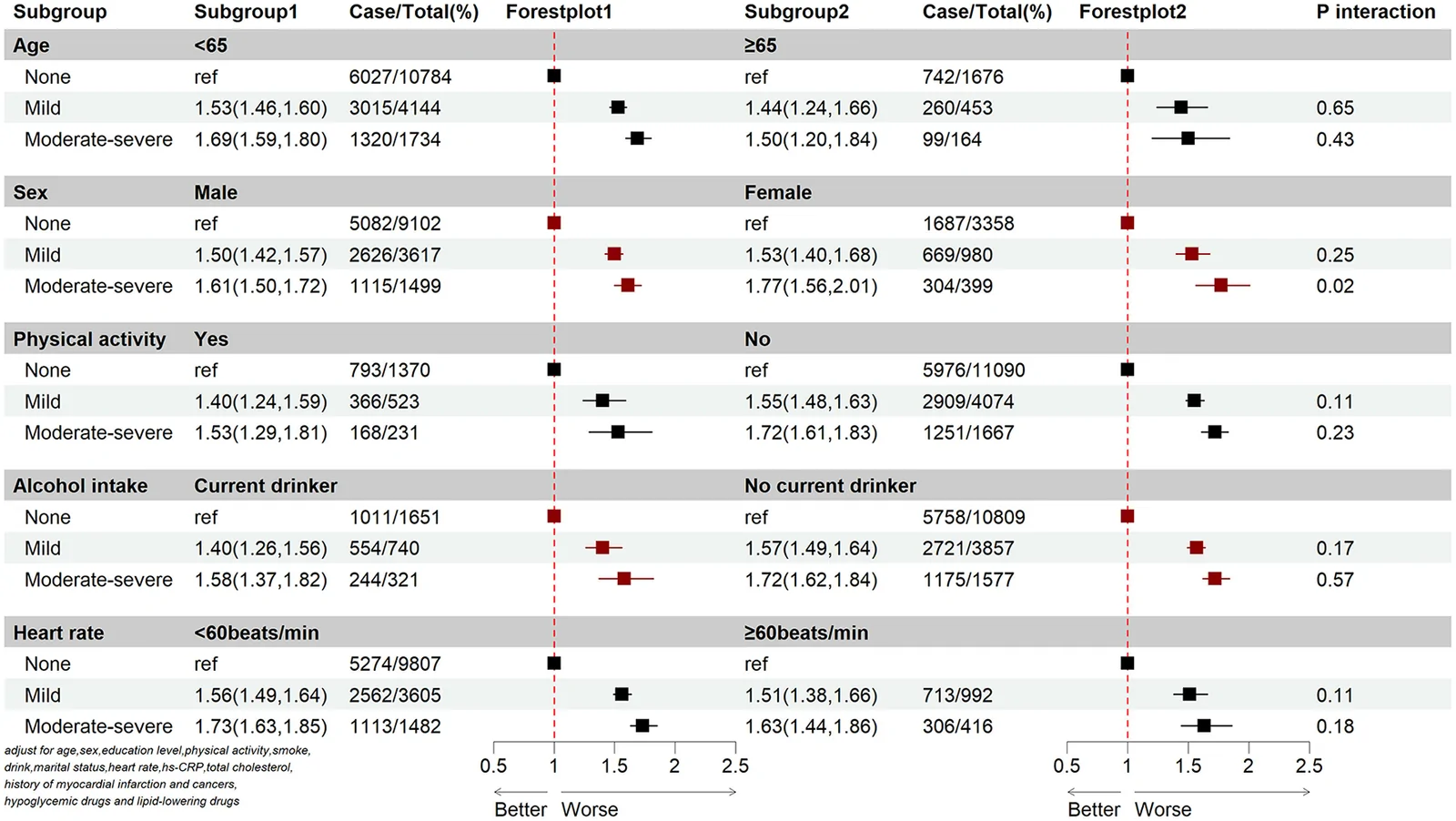
Stratified analyses by fatty liver severity for incident clinical obesity. Forest plot shows hazard ratios (HRs) and 95% confidence intervals (CIs) for baseline mild fatty liver and moderate-to-severe fatty liver (each compared to those without fatty liver) across various subgroups (age, sex, physical activity, alcohol intake, and heart rate). The HRs are presented for individuals aged <65 and ≥65 years, male and female, with and without physical activity, current drinkers and non-drinkers, and different heart rate categories (<60 and ≥60 beats per minute). Horizontal bars represent 95% CIs, and squares indicate point estimates. The vertical dashed line corresponds to HR = 1.0. *P* for interaction was calculated from multiplicative interaction terms in Cox models. CI, confidence interval; HR, hazard ratio; hs-CRP, high-sensitivity C-reactive protein.

### Distribution of organ dysfunction in individuals progressing from preclinical to clinical obesity

To further investigate the impact of fatty liver on progression from preclinical to clinical obesity, we analyzed the distribution, types, and proportions of organ dysfunction in individuals with clinical obesity. As shown in Figure 6a, among the 11,463 individuals who developed clinical obesity, the proportion of participants with one to five affected organ systems was 50.87%, 37.77%, 10.56%, 0.79%, and 0.02%, respectively. The three most common types of single organ dysfunction were hypertension (84.2%, *n* = 9,649), obstructive sleep apnea syndrome (OSAS; 33.9%, *n* = 3,888), and hyperglycemia-dyslipidemia syndrome (27.9%, *n* = 3,197). The most common combinations of multiple organ dysfunctions were: Hypertension with OSAS (18.2%, *n* = 2,090), hypertension with hyperglycemia-dyslipidemia syndrome (13.7%, *n*=1,570), and the combination of hypertension, hyperglycemia-dyslipidemia syndrome, and OSAS (7.1%, *n*=809; Figure 6b). Further analysis revealed that in individuals with clinical obesity and concomitant fatty liver, the burden of organ dysfunction was more evenly distributed: 44.35% had one organ dysfunction and 41.39% had two organ dysfunctions (Supplementary Figure S1). The most common types of organ dysfunction, whether single or multiple, were consistent with the general patterns observed in the overall clinical obesity cohort (Supplementary Figure S2).

**Figure 6:**
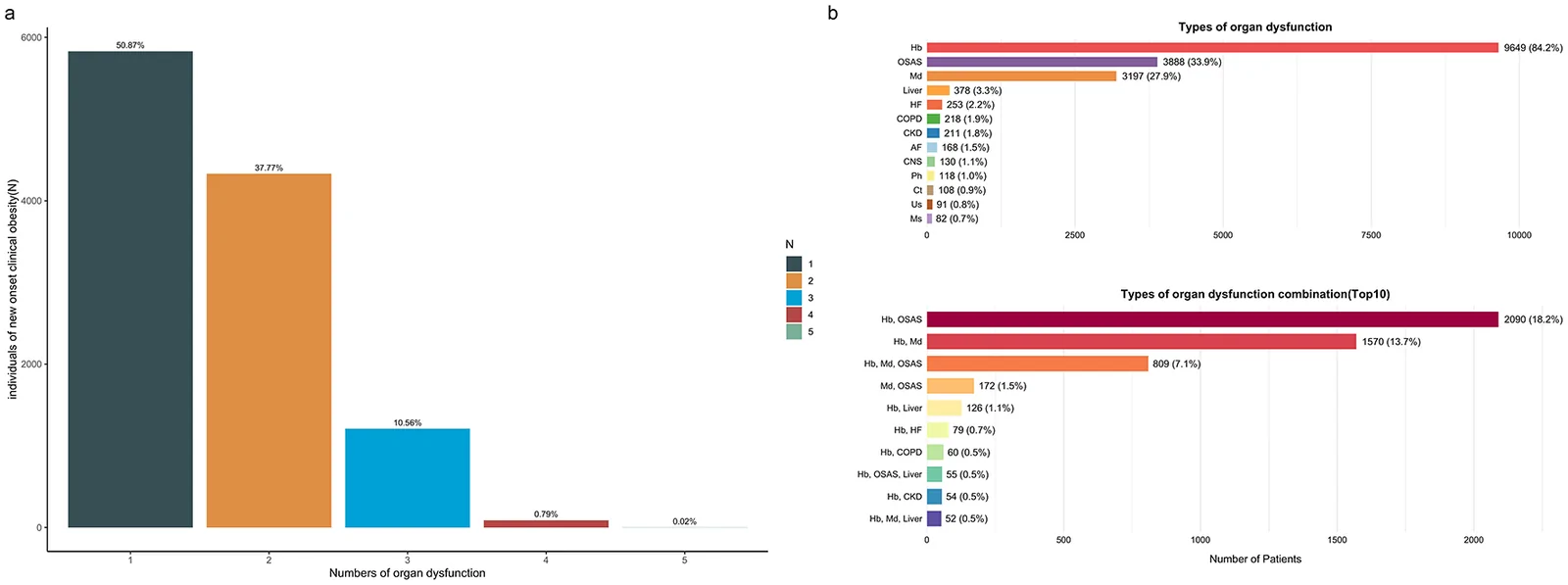
Obesity-related organ dysfunctions among participants with incident clinical obesity. **(a)** Distribution of incident clinical obesity by the number of organ dysfunctions. Most participants had 1 dysfunction (50.87%) or 2 dysfunctions (37.77%), whereas 3 (10.56%), 4 (0.79%) or 5 (0.02%) dysfunctions were uncommon. **(b)** Types of organ dysfunction and the most frequent combinations in incident clinical obesity. The upper panel shows the frequency of each dysfunction type; hypertension (Hb, 84.2%) and obstructive sleep apnea syndrome (OSAS, 33.9%) were most common, followed by metabolic dysfunction (Md, 27.9%). The lower panel presents the top 10 combinations, with Hb + OSAS (18.2%) and Hb + Md (13.7%) being the most frequent; percentages are shown next to bars. Hb, hypertension; OSAS, obstructive sleep apnea syndrome; Md, metabolic dysfunction; HF, heart failure; COPD, chronic obstructive pulmonary disease; CKD, chronic kidney disease; AF, atrial fibrillation; CNS, central nervous system; Ph, pulmonary hypertension; Ct, cardiovascular thrombosis; Us, urinary system; Ms, musculoskeletal system.

## Discussion

In this prospective cohort study involving approximately 100,000 Chinese participants from the Kailuan population, we demonstrated for the first time that preclinical obesity combined with ultrasound-defined fatty liver is significantly associated with an increased risk of progressing to clinical obesity. Importantly, this association exhibited a clear relationship with increasing severity of fatty liver disease and was found to differ by sex, particularly among participants with moderate-to-severe fatty liver. Additionally, among those who progressed to clinical obesity, single-organ dysfunction was most prevalent, with hypertension, OSAS, and hyperglycemia-dyslipidemia syndrome emerging as the predominant clinical conditions.

To our knowledge, this is the first prospective cohort study to investigate the risk factors for progression from preclinical obesity to clinical obesity. Although previous cross-sectional studies have suggested an association between fatty liver and obesity (28–30), longitudinal data were lacking to determine whether fatty liver serves as a precursor or risk factor for obesity-related complications. Compared to the obesity definition recently proposed by the Lancet Diabetes and Endocrinology Commission, the European Association for the Study of Obesity (EASO) introduced a new diagnostic criterion in 2024 that includes BMI, WtHR and at least one of the five major obesity-related complications (diabetes, hypertension, CVD, MetS and accelerated aging) (31). This approach overcomes the limitations of BMI alone. Recent studies using these new criteria in the US population showed an increased prevalence of obesity and improved prediction of all-cause, cardiovascular and cancer mortality compared to traditional BMI-based definitions (32). However, relevant studies in Asian populations remain scarce, especially those using clinical obesity as an endpoint. In this study, we found that individuals with preclinical obesity accompanied by ultrasound-defined fatty liver had a significantly increased risk of developing clinical obesity, with the risk increasing as the severity of fatty liver progressed. These findings not only confirm for the first time a positive association between fatty liver and the risk of clinical obesity in the Chinese and broader East Asian population, but also support the interpretation of ultrasound-defined fatty liver as an early hepatic manifestation of metabolic dysfunction during the progression from preclinical to clinical obesity.

Importantly, ultrasound-defined fatty liver may represent an early and distinct hepatic manifestation of established metabolic dysfunction in individuals classified as having preclinical obesity. This perspective extends clinical assessment beyond BMI and anthropometric indices, suggesting that hepatic changes may precede overt obesity-related organ dysfunction and that excess adiposity may partly reflect underlying metabolic impairment. Accordingly, fatty liver may indicate a potentially reversible metabolic abnormality warranting earlier metabolic assessment and intervention.

Consistent with this concept, the progression from preclinical obesity to clinical obesity is characterized by concomitant organ dysfunction. In particular, MetS shares several diagnostic components with the organ dysfunction observed in clinical obesity, including abdominal obesity, hyperglycemia, hypertension, and dyslipidemia. An earlier cohort study from Korea showed a positive association between fatty liver and the occurrence of MetS. This study, which included a 5-year follow-up of 2,089 participants without hypertension, diabetes or MetS at baseline, showed that persistent fatty liver status significantly increased the risk of developing MetS (33). Another analysis of the same cohort, which included 11,448 participants without hypertension at baseline, showed that new-onset fatty liver was positively associated with the risk of developing hypertension during 5-year follow-up (adjusted odds ratio [aOR] = 1.60), an association that remained significant after adjustment for BMI (34). In addition, previous studies have shown that the risk of diabetes increases with the severity of fatty liver from mild to moderate/severe (35). In our study, we observed a positive association between preclinical obesity in combination with fatty liver and the risk of clinical obesity. In line with the findings on MetS, hypertension, dysglycemia and dyslipidemia were identified as the main organ dysfunctions associated with clinical obesity. In addition, we found that the risk of progression to clinical obesity in individuals of preclinical obesity with fatty liver gradually decreased over time, but remained high overall. Interestingly, this downward trend differed according to the severity of fatty liver: individuals with moderate-to-severe fatty liver had a higher risk of progressing to clinical obesity during the first 10 years of follow-up than those with mild fatty liver. After 10 years, however, individuals with a mild fatty liver showed a higher relative risk. The reasons for these different time-varying risk patterns remain unclear. Earlier progression among participants with more severe fatty liver, subsequent clinical management, changes in hepatic steatosis, and other unmeasured factors may have contributed to this pattern. However, these possibilities could not be evaluated because longitudinal data on treatment, inflammatory status, insulin sensitivity, and changes in hepatic steatosis were unavailable. Further longitudinal and mechanistic studies are required to clarify the underlying mechanisms. Nevertheless, both groups showed a consistently increased overall risk, emphasizing the importance of early identification and management of fatty liver to mitigate the progression from preclinical to clinical obesity.

The accumulation of lipids in non-obese tissues is a hallmark of the progression of obesity-related disease, including atherosclerosis, type 2 diabetes mellitus and MASLD (36). Although the concept of lipotoxicity has been known for many years, the exact relationship between excessive fat deposition and organ dysfunction remains an area of research. Previous studies have indicated that fatty liver increases the strength of the association between MetS and type 2 diabetes (37). However, another study has reported that MHO individuals have an increased incidence of diabetes regardless of the presence of NAFLD, suggesting that the obese phenotype itself may independently contribute to diabetes risk (38). In the present study, we found that hypertension, OSAS, and hyperglycemia-dyslipidemia syndrome were the predominant forms of organ dysfunction observed in participants with clinical obesity, regardless of whether they had single or multiple organ dysfunctions. In addition, individuals with ultrasound-defined fatty liver had a higher incidence of hypertension and hyperglycemia-dyslipidemia syndrome and a higher likelihood of multiple organ dysfunctions than those without fatty liver. These findings emphasize the importance of strengthening preventive strategies and clinical management of fatty liver, including weight control, dietary measures and promotion of physical activity. In addition, proactive management of blood pressure, glucose and lipid profiles is essential to effectively reduce the incidence and impact of clinical obesity. In contrast, in individuals of preclinical obesity without fatty liver, special attention should be paid to the early detection and management of OSAS in addition to blood pressure monitoring.

Previous studies have suggested sex differences in the metabolic consequences of abdominal obesity and fatty liver. In a cross-sectional study of 9,685 Chinese adults, abdominal obesity was more strongly associated with insulin resistance and hypertension in men, whereas fatty liver showed stronger associations with elevated fasting glucose and hypertriglyceridemia in women (39). Consistent with these observations, our exploratory subgroup analyses suggested that the association of fatty liver, particularly moderate-to-severe fatty liver, with incident clinical obesity may be stronger in women.. Although women generally exhibit relative protection against ectopic fat accumulation and cardiometabolic dysfunction, particularly before menopause, this protection may diminish during and after the menopausal transition (40). Therefore, our findings should not be interpreted as indicating a higher absolute risk of clinical obesity in women; rather, hepatic steatosis may identify a metabolically vulnerable subgroup of women in whom this relative protection has been attenuated. However, menopausal status and sex hormone levels were unavailable and the number of female participants was relatively limited.. Exploratory analyses also suggested that the association between fatty liver and incident clinical obesity may be stronger among participants with a resting heart rate ≥60 bpm. This pattern, however, was not consistently observed in the analyses stratified by fatty liver severity. A possible explanation is that elevated resting heart rate reflects autonomic nervous system dysregulation that may interact with underlying metabolic abnormalities. The inconsistent findings across severity categories may also be attributable to limited statistical power, pathologic heterogeneity, or residual confounding. Moreover, because multiple interaction tests were conducted without adjustment for multiple comparisions, the observed sex- and heart rate-related interactions were based on nominal *P* values and should be consider exploratory. Future studies with larger and more sex-balanced populations, detailed reproductive and hormonal information, and independent validation are need.

The main strength of this study lies in its novel approach as the first large-scale prospective cohort to systematically investigate risk factors associated with the transition from preclinical to clinical obesity. Our results provide robust evidence that ultrasound-defined fatty liver is an independent risk factor significantly associated with the onset of clinical obesity. The longitudinal study revealed a characteristic spectrum of organ dysfunction, with hypertension, OSAS, and hyperglycemia-dyslipidemia syndrome identified as the central triad underlying clinical obesity-related organ dysfunction. However, some limitations of our study must be acknowledged. First, the diagnosis of fatty liver was primarily based on ultrasound imaging, the accuracy of which depends largely on the experience of the ultrasonographers, potentially leading to examiner bias. Moreover, inter-rater reliability was not formally quantified in this study, which may limit the assessment of consistency across examiners. Future studies should utilize additional diagnostic methods such as magnetic resonance imaging or liver biopsy to confirm the presence and severity of fatty liver and incorporate standardized reliability assessments to improve diagnostic accuracy and research credibility. Second, the diagnostic criteria proposed by the 2025 Lancet Commission were retrospectively operationalized using data collected from 2006 onward. Because the original examinations were not specifically designed for this contemporary framework, some organ dysfunctions may have been incompletely captured, and the available historical variables may not fully correspond to the current diagnostic criteria. In particular, liver fibrosis was assessed using FIB-4 rather than elastography or histology. Although FIB-4 may overestimate fibrosis risk in older adults, only 62 participants were excluded on this basis, and their re-inclusion did not materially alter the results. Nevertheless, residual misclassification of preclinical obesity and incident clinical obesity cannot be excluded. Third, preclinical obesity was defined using alternative and overlapping anthropometric criteria, resulting in a potentially heterogeneous population. Consequently, we could not determine whether the association differed across the individual criteria, and potential heterogeneity-related bias cannot be excluded. Fourth, although an association between fatty liver and the occurrence of clinical obesity has been demonstrated, causality could not be established from the available observational data. Therefore, future research with genetic analyses or molecular studies is needed to clarify possible causal relationships and the underlying biological mechanisms. Fifth, fatty liver was assessed only at baseline and was not reassessed during follow-up. Therefore, given its dynamic and potentially reversible nature, longitudinal changes in fatty liver status or severity, including persistence, resolution, and progression, could not be evaluated. Therefore, we were unable to determine how longitudinal changes in fatty liver status or severity influenced the progression from preclinical to clinical obesity. Future studies incorporating repeated assessments of fatty liver are needed to address this issue. Finally, this study was conducted primarily among residents of the Kailuan community in northern China, and it was a predominantly male cohort. Future large-scale prospective studies with more geographically and ethnically diverse populations and sex balance are warranted to generalize and confirm our findings.

## Conclusions

In summary, baseline ultrasound-defined fatty liver was associated with an increased risk of progression from preclinical to clinical obesity, with stronger associations observed at greater fatty liver severity. Hypertension, OSAS and hyperglycemia-dyslipidemia syndrome were the most common forms of organ dysfunction among participants who developed clinical obesity. These findings suggest that ultrasound-defined fatty liver detected during routine examinations may provide a practical imaging phenotype for risk stratification among individuals with preclinical obesity and may help identify those who warrant closer monitoring of blood pressure, glucose and lipid profiles. Further studies are needed to determine whether incorporating liver ultrasonography into targeted risk-stratification strategies can improve subsequent clinical outcomes.

## Data Availability

The raw data supporting the conclusions of this article will be made available by the authors, without undue reservation.

## Supplementary material

Supplementary Figure S1 Presence of fatty liver and proportion of incident clinical obesity-related organ dysfunction types. The picture on the left shows the group without fatty liver, and the picture on the right shows the group with fatty liver.

Supplementary Figure S2 The presence or absence of fatty liver and the specific types and distribution of incident clinical obesity-related organ dysfunction. The top three proportions of incident clinical obesity-related single organ dysfunction in the group without fatty liver are hypertension, OSAS, hyperglycemia and dyslipidemia syndrome. The top three incidences of new clinical obesity-related multiple organ dysfunction in the group without fatty liver are hypertension-OSAS, hypertension-hyperglycemia and dyslipidemia, hypertension-hyperglycemia and dyslipidemia-OSAS. The top three proportions of incident clinical obesity-related single organ dysfunction in the group with fatty liver are hypertension, hyperglycemia and dyslipidemia syndrome, OSAS. The top three incidences of new clinical obesity-related multiple organ dysfunction in the group with fatty liver are hypertension-hyperglycemia and dyslipidemia, hypertension-OSAS, hypertension-hyperglycemia and dyslipidemia-OSAS).
