# Supplementary material for: Ultrasound-defined fatty liver and the risk of progression from preclinical to clinical obesity: a prospective cohort study
Source: Front Nutr. 2026 Sep 22;13:1877518. doi: 10.3389/fnut.2026.1877518 (PMC13638645; doi:10.3389/fnut.2026.1877518)
Supplement: Supplementary file 1 [file Table_1.docx]

**Table S1** Anthropometric Indicators Criteria

| **Indicator** | **Calculation Formula** | **Risk Threshold** |
| --- | --- | --- |
| BMI (kg/m²) | Weight (kg) / Height (m²) | >40  ≥28 |
| Waist Circumference (cm) | – | ≥90 (Male) ≥85 (Female) |
| WHtR | Waist Circumference(cm) / Height (cm) | ≥0.50 |
| WHR | Waist Circumference (cm) / Hip (cm) | ≥0.90 (Male) ≥0.85 (Female) |
