# Supplementary material for: Ultrasound-defined fatty liver and the risk of progression from preclinical to clinical obesity: a prospective cohort study
Source: Front Nutr. 2026 Sep 22;13:1877518. doi: 10.3389/fnut.2026.1877518 (PMC13638645; doi:10.3389/fnut.2026.1877518)
Supplement: Supplementary file 2 [file Table_2.docx]

**Table S2** Diagnostic criteria for clinical obesity in Kailuan Study

| **Types of Organ dysfunction** | **Diagnostic criteria** |
| --- | --- |
| **Cardiovascular (arterial)** | SBP ≥140 mmHg or DBP ≥90 mmHg, use of any antihypertensive agents, or self-reported history of hypertension |
| **Metabolism** | Syndrome of hyperglycemia (FBG≥6.1 mmol/L or type 2 diabetes according to ADA criteria) combined with dyslipidemia (TG ≥1.7 mmol/L or HDL <1.0 mmol/L) |
| **Upper airways** | Patients with a confirmed diagnosis of obstructive sleep apnea or persistent snoring |
| **Respiratory** | Patients with a discharge diagnosis of chronic obstructive pulmonary disease |
| **Cardiovascular system (atrial)** | ECG-confirmed atrial fibrillation |
| **Cardiovascular system (ventricular)** | Heart failure in accordance with the Chinese Guidelines for the Diagnosis and Treatment of Heart Failure[5] |
| **Cardiovascular (thrombosis)** | Recurrent Deep vein thrombosis and/or pulmonary thromboembolism confirmed by imaging |
| **Renal** | Using the CKD-EPI formula estimates eGFR, defined as eGFR <60mL/min/1.73m^2^ and urine protein 2(+) measured by urinalysis. |
| **Cardiovascular (pulmonary)** | Cardiac ultrasound estimates PASP by measuring tricuspid regurgitation velocity. If PASP ≥40mmHg, it indicates possible pulmonary hypertension |
| **Musculoskeletal** | Knee or hip osteoarthritis |
| **Urinary** | Recurrent/chronic urinary incontinence |
| **Liver** | NAFLD with liver fibrosis: NAFLD is diagnosed through liver ultrasound and drinking status. Liver fibrosis is assessed by using FIB-4 score. FIB-4 ≥3.25 indicates significant liver fibrosis or cirrhosis. |
| **CNS** | The discharge diagnosis is increased intracranial pressure or recurrent headaches, excluding concurrent acute cerebral infarction, cerebral hemorrhage, brain trauma and encephalitis. |
| SBP: systolic blood pressure; DBP: diastolic blood pressure; TG: triglycerides; FBG: fasting blood glucose;  HDL: high-density lipoprotein; CKD-EPI: Chronic Kidney Disease Epidemiology Collaboration;  PASP: pulmonary artery systolic pressure; eGFR: glomerular filtration rate;  NAFLD: Non-alcoholic fatty liver disease; CNS: Central nervous system | |

For chronic diseases requiring long-term management and lacking definitive cures, outpatient care strategies are designed to reduce patient financial strain and refine therapeutic optimization. To enhance diagnostic validity, this study integrated clinical diagnostic data with disease-specific diagnostic patterns and pharmacotherapeutic records derived from outpatient chronic disease registries.
