# Supplementary material for: Ultrasound-defined fatty liver and the risk of progression from preclinical to clinical obesity: a prospective cohort study
Source: Front Nutr. 2026 Sep 22;13:1877518. doi: 10.3389/fnut.2026.1877518 (PMC13638645; doi:10.3389/fnut.2026.1877518)
Supplement: Supplementary file 3 [file Table_3.docx]

**Table S3** Multicollinearity screening of covariates

| **Variable** | **VIF** |
| --- | --- |
| Age | 1.138 |
| Sex | 1.150 |
| Marital status | 1.026 |
| Education level | 1.150 |
| Physical activity | 1.073 |
| Current smoking  Current alcohol consumption | 1.376  1.251 |
| Heart rate | 1.011 |
| hs-CRP  TC | 1.013  1.049 |
| Hypoglycemic medication | 1.013 |
| Lipid-lowering medication | 1.068 |
| History of MI  History of cancer | 1.009  1.005 |
| Abbreviations: MI, myocardial infarction; TC, total cholesterol. | |
