# Supplementary material for: Ultrasound-defined fatty liver and the risk of progression from preclinical to clinical obesity: a prospective cohort study
Source: Front Nutr. 2026 Sep 22;13:1877518. doi: 10.3389/fnut.2026.1877518 (PMC13638645; doi:10.3389/fnut.2026.1877518)
Supplement: Supplementary file 4 [file Table_4.docx]

| **Table S4**  Time-varying association of baseline fatty liver status with incident clinical obesity among participants with preclinical obesity | | | | |
| --- | --- | --- | --- | --- |
|  | **Without fatty liver** | **Fatty liver** | **Rate of change in HR/per year** | |
| Model 1 | 1(reference) | 1.84(1.72-1.97) | | -2.1% |
| Model 2 | 1(reference) | 1.77(1.65-1.89) | | -1.9% |
| Model 3 | 1(reference) | 1.74(1.62-1.86) | | -1.9% |
| HRs were estimated using extended Cox models with a time-varying coefficient for baseline fatty liver status. Fatty liver status and all covariates were assessed at baseline and were not updated during follow-up. The reported HRs represent the estimated associations at the end of follow-up. Negative values for annual relative change indicate attenuation of the HR over time. Model 1: adjusted for age sex; Model 2: adjust for education level, physical activity, current smoke, current drink, marital status, heart rate, hs-CRP, total cholesterol on the basis of model 1; Model 3: included variables in model 2 and further adjusted for history of myocardial infarction and cancers, hypoglycemic drugs and lipid-lowering agents. Abbreviations: CI, confidence interval; HR, hazard ratio. | | | | |
