# Supplementary material for: Ultrasound-defined fatty liver and the risk of progression from preclinical to clinical obesity: a prospective cohort study
Source: Front Nutr. 2026 Sep 22;13:1877518. doi: 10.3389/fnut.2026.1877518 (PMC13638645; doi:10.3389/fnut.2026.1877518)
Supplement: Supplementary file 5 [file Table_5.docx]

**Table S5** Time-varying associations of baseline fatty liver severity with incident clinical obesity among participants with preclinical obesity

|  | **Without fatty liver** | **Fatty liver** | | | |
| --- | --- | --- | --- | --- | --- |
|  |  | **Mild** | **Rate of change in HR/per year** | **Moderate-Severe** | **Rate of change in HR/per year** |
| Model1 | 1(reference) | 1.74(1.62-1.88) | -1.7% | 2.09(1.88-2.32) | -3.2% |
| Model2 | 1(reference) | 1.67(1.55-1.81) | -1.4% | 2.00(1.81-2.23) | -3.0% |
| Model3 | 1(reference) | 1.65(1.53-1.78) | -1.4% | 1.96(1.76-2.18) | -3.0% |
| HRs were estimated using extended Cox models with time-varying coefficients for baseline fatty liver severity. Fatty liver severity and all covariates were assessed at baseline and were not updated during follow-up. Participants without fatty liver at baseline served as the reference group. The reported HRs represent the estimated associations at the end of follow-up. Negative values for annual relative change indicate attenuation of the HR over time. Model 1: adjusted for age sex; Model 2: adjust for education level, physical activity, current smoke, current drink, marital status, heart rate, hs-CRP, total cholesterol on the basis of model 1; Model 3: included variables in model 2 and further adjusted for history of myocardial infarction and cancers, hypoglycemic drugs and lipid-lowering agents. Abbreviations: CI, confidence interval; HR, hazard ratio. | | | | | |
