# Supplementary material for: Ultrasound-defined fatty liver and the risk of progression from preclinical to clinical obesity: a prospective cohort study
Source: Front Nutr. 2026 Sep 22;13:1877518. doi: 10.3389/fnut.2026.1877518 (PMC13638645; doi:10.3389/fnut.2026.1877518)
Supplement: Supplementary file 6 [file Table_6.docx]

| **Table S6** Sensitivity analysis of excluding outcomes within the first year of follow-up (event=3 total=18952) | | | |
| --- | --- | --- | --- |
|  | **Without fatty liver** | **Fatty liver** | |
|  |  | **Mild** | **Moderate- Severe** |
| Case/Total  IR | 6768/12459  61.11 | 3273/4595  100.70 | 1419/1898  112.34 |
|  | 1 (Ref.) | 1.54(1.47-1.61) | 1.70(1.60-1.80) |
| Note: IR，incidence rate (per 1000 person-years); Adjusted for age, sex, heart rate, total cholesterol, hs-CRP, current smoker, current drinker, physical activity, education level, hypoglycemic drugs and lipid-lowering agents, history of myocardial infarction, history of cancers | | | |
