# Supplementary material for: Ultrasound-defined fatty liver and the risk of progression from preclinical to clinical obesity: a prospective cohort study
Source: Front Nutr. 2026 Sep 22;13:1877518. doi: 10.3389/fnut.2026.1877518 (PMC13638645; doi:10.3389/fnut.2026.1877518)
Supplement: Supplementary file 7 [file Table_7.docx]

| **Table S7** Sensitivity analysis of excluding individuals with the use of hypoglycemic drugs or lipid-lowering agents, respectively | | | |
| --- | --- | --- | --- |
|  | **Without fatty liver** | **Fatty liver** | |
|  |  | **Mild** | **Moderate- Severe** |
| **Excluding individuals with the use of Hypoglycemic agents(n=209)** | | | |
| Case/Total  IR | 6688/12338  60.89 | 3231/4535  100.74 | 1403/1873  112.77 |
|  | 1 (Ref.) | 1.54(1.48-1.61) | 1.71(1.62-1.82) |
| **Excluding participants with the use of Lipid-lowering drugs(n=6275)** | | | |
| Case/Total  IR | 4726/9009  57.83 | 1870/2712  94.49 | 702/959  106.15 |
|  | 1 (Ref.) | 1.57 (1.49-1.66) | 1.75(1.61-1.90) |
| Note: IR，incidence rate (per 1000 person-years); Adjusted for age, sex, heart rate, total cholesterol, hs-CRP, current smoker, current drinker, physical activity, education level, hypoglycemic drugs and lipid-lowering agents, history of myocardial infarction, history of cancers | | | |
