# Supplementary material for: Ultrasound-defined fatty liver and the risk of progression from preclinical to clinical obesity: a prospective cohort study
Source: Front Nutr. 2026 Sep 22;13:1877518. doi: 10.3389/fnut.2026.1877518 (PMC13638645; doi:10.3389/fnut.2026.1877518)
Supplement: Supplementary file 8 [file Table_8.docx]

| **Table S8** Sensitivity analysis excluding participants with BMI < 24 (n=5216). | | | |
| --- | --- | --- | --- |
|  | **Without fatty liver** | **Fatty liver** | |
|  |  | **Mild** | **Moderate- Severe** |
| Case/Total | 4955/7869 | 2993/4110 | 1360/1793 |
| IR | 77.94 | 105.49 | 115.64 |
|  | 1 (Ref.) | 1.31 (1.25-1.37) | 1.41 (1.33-1.50) |

Note: IR，incidence rate (per 1000 person-years); Adjusted for age, sex, heart rate, total cholesterol, hs-CRP, current smoker, current drinker, physical activity, education level, hypoglycemic drugs and lipid-lowering drugs, history of myocardial infarction, history of cancers
