# Supplementary material for: Ultrasound-defined fatty liver and the risk of progression from preclinical to clinical obesity: a prospective cohort study
Source: Front Nutr. 2026 Sep 22;13:1877518. doi: 10.3389/fnut.2026.1877518 (PMC13638645; doi:10.3389/fnut.2026.1877518)
Supplement: Supplementary file 9 [file Table_9.docx]

| **Table S9** Sensitivity analysis re-inclusion of the 62 participants originally excluded because of a FIB-4 score > 3.25. | | | |
| --- | --- | --- | --- |
|  | **Without fatty liver** | **Fatty liver** | |
|  |  | **Mild** | **Moderate- Severe** |
| Case/Total | 6802/12512 | 3280/4602 | 1420/1899 |
| IR | 61.18 | 100.84 | 112.33 |
|  | 1 (Ref.) | 1.54 (1.48-1.61) | 1.70 (1.60-1.80) |

Note: IR，incidence rate (per 1000 person-years); Adjusted for age, sex, heart rate, total cholesterol, hs-CRP, current smoker, current drinker, physical activity, education level, hypoglycemic drugs and lipid-lowering drugs, history of myocardial infarction, history of cancers
